# Supplementary material for: Novel ATP-cone-driven allosteric regulation of ribonucleotide reductase via the radical-generating subunit
Source: eLife. 2018 Feb 1;7:e31529. doi: 10.7554/eLife.31529 (PMC5794259; doi:10.7554/eLife.31529)
Supplement: Figure 6—source data 1. — Figures in parentheses are for the highest resolution shell. [file elife-31529-fig6-data1.docx]

**Supplementary Table S1:** Data quality statistics for the anomalous data used for metal identification.Figures in parentheses are for the highest resolution shell.

| **Wavelength** | 1.7200 | 1.8700 | 1.9200 |
| --- | --- | --- | --- |
| **Resolution range** | 79.7-2.50 (2.56-2.50) | 74.1-2.50 (2.56-2.50) | 74.1–2.50 (2.56-2.50) |
| **Space group** | P1 | | |
| **Unit cell (Å, °)** | a = 74.5, b = 91.5, c = 91.3,  α = 60.8, β = 66.4, γ = 80.6 | a = 74.6, b = 91.4, c = 91.4,  α = 60.8, β = 66.3, γ = 80.6 | a = 74.4, b = 91.3, c = 91.3,  α = 60.8, β = 66.3, γ = 80.6 |
| **Total no. reflections** | 233102 (17171) | 229234 (16493) | 230917 (17003) |
| **Unique reflections** | 60658 (4421) | 59917 (4374) | 59645 (4542) |
| **Anomalous multiplicity** | 2.0 (2.0) | 2.0 (1.9) | 2.0 (1.9) |
| **Completeness (%)** | 90.9 (89.4) | 86.0 (84.2) | 85.8 (82.1) |
| **Mean I/σ(I)** | 11.8 (0.9) | 12.9 (1.5) | 11.5 (0.6) |
| **R_merge_ (I)** | 0.056 (1.222) | 0.051 (0.750) | 0.065 (1.771) |
| **R_meas_ (I)** | 0.065 (1.416) | 0.060 (0.875) | 0.076 (2.072) |
| **R_pim_ (I)** | 0.033 (0.714) | 0.031 (0.448) | 0.038 (1.070) |
| **CC(½) (I)** | 0.999 (0.633) | 0.999 (0.758) | 0.999 (0.423) |
