# Supplementary material for: Novel ATP-cone-driven allosteric regulation of ribonucleotide reductase via the radical-generating subunit
Source: eLife. 2018 Feb 1;7:e31529. doi: 10.7554/eLife.31529 (PMC5794259; doi:10.7554/eLife.31529)
Supplement: Figure 6—source data 2. — The peak heights are given in standard deviations above the mean. For reference, the anomalous difference map peak heights are also given for two of the most well-ordered sulfur atoms in the structure, in Met A80 and Met D32. It can be seen that the average peak heights for the metal ions vary little with wavelength and are at most only about twice the height of those for the sulfur atoms, which is most consistent with them being Ca2+, whose f’ component is about twice that of sulfur at all three wavelengths, whereas that of Mn2+ or Fe2+/3+ is more than four times higher at 1.72 Å and 1.87 Å. [file elife-31529-fig6-data2.docx]

**Supplementary Table S2:** Peak heights of metal ions in anomalous difference maps calculated at three different wavelengths after refining the same structure against data at each wavelength. The peak heights closest to the given metal ion positions are given in standard deviations above the mean. For reference, the anomalous difference map peak heights are also given for two of the most well-ordered sulfur atoms in the structure, in Met A80 and Met D32. The first table reports maps calculated at 2.5 Å resolution, the second at 4.0 Å resolution. It can be seen that the average peak heights for the metal ions vary little with wavelength and are at best only about twice the height of those for the sulfur atoms, which is most consistent with them being Ca^2+^, whose f” component is about twice that of sulfur at all three wavelengths, whereas that of Mn^2+^ or Fe^2+/3+^ is more than four times higher.

| wavelength | **1.72 Å** | **1.87 Å** | **1.92 Å** |
| --- | --- | --- | --- |
| **A1004** | 8.6 | 10.9 | 8.5 |
| **A1005** | 10.9 | 12.3 | 8.6 |
| **B1004** | 11.9 | 12.4 | 9.1 |
| **B1005** | 9.2 | 10.6 | 9.2 |
| **C1004** | 10.2 | 10.3 | 6.5 |
| **C1005** | 6.2 | 9.0 | 6.7 |
| **D1004** | 12.0 | 9.8 | 7.9 |
| **D1005** | 10.8 | 10.4 | 8.3 |
| **average(Me)** | 10.0 | 10.7 | 8.1 |
| **SD MetA80** | 5.1 | 5.0 | 5.2 |
| **SD MetD32** | 4.9 | 5.1 | 4.6 |

| wavelength | **1.72 Å** | **1.87 Å** | **1.92 Å** |
| --- | --- | --- | --- |
| **A1004** | 9.2 | 9.6 | 9.7 |
| **A1005** | 10.2 | 9.6 | 9.6 |
| **B1004** | 10.5 | 10.1 | 10.2 |
| **B1005** | 8.1 | 10.1 | 10.2 |
| **C1004** | 10.3 | 8.4 | 8.1 |
| **C1005** | 6.0 | 6.0 | 8.7 |
| **D1004** | 9.6 | 8.8 | 8.7 |
| **D1005** | 9.9 | 10.9 | 9,5 |
| **average(Me)** | 9.2 | 9.3 | 9.3 |
| **SD MetA80** | 5.6 | 4.7 | 5.5 |
| **SD MetD32** | 5.5 | 5.9 | 5.5 |
